# A case of *Citrobacter koseri* renal abscess and review of the literature

**DOI:** 10.1177/2050313X221135347

**Published:** 2022-11-01

**Authors:** Duong Tommy Hua, Jessica Lo, Huy Quang Do, Charles Dac Pham

**Affiliations:** Department of Internal Medicine, Harbor-UCLA Medical Center, Torrance, CA, USA

**Keywords:** Infectious diseases, *Citrobacter koseri*, renal abscess, kidney abscess

## Abstract

*Citrobacter* species are anaerobic gram-negative bacteria that are known to cause infections in immunocompromised hosts, particularly in hospital settings. Their opportunistic nature and tendency to develop antibiotic resistance make *Citrobacter* species challenging to treat. Renal or perinephric abscess formation as a result of *Citrobacter* infection is uncommon, having only previously been reported in four cases. We present a case of a 70-year-old man with diabetes and prostate cancer who was diagnosed with an 18 cm perinephric and a 10 cm perihepatic abscess caused by *Citrobacter koseri*. The patient required drains and re-positioning of the drains multiple times in addition to a prolonged course of antibiotics to achieve complete radiographic resolution. This case highlights the challenges in treating renal and perinephric abscess, as it required drain re-placements two additional times after the initial placement and an additional 4 weeks of antibiotics. Successful treatment for larger abscesses usually requires a two-arm approach, with antibiotics in combination with either percutaneous or surgical abscess drainage.

## Introduction

*Citrobacter* bacteria are opportunistic anaerobic gram-negative rods that can be found in the intestinal tracts of both animals and humans, and in soil, water, and food.^1,2^ *Citrobacter* species can cause infections of the urinary tract, respiratory tract, intra-abdominal organs, skin, and soft tissues, and are also known to cause bacteremia, endocarditis, osteomyelitis, and meningitis.^1,3,4^ In one study of 205 patients with *Citrobacter* infections, 46.2% of cases were isolated in the urine, 16.3% in the respiratory tract, 15.8% in the blood, and 4.3% in wounds.^3^ *C. koseri* and *C. freundii* account for most of the infections. Greater than 80% of patients with *Citrobacter* infections have an underlying medical condition, such as diabetes, cardiac disease, pulmonary disease, renal disease, hepatobiliary disease, neurologic disease, malignancy, or structural abnormalities of the urinary tract.^3,4^

*C. koseri* abscess formation is uncommon, and has been described in the kidney, muscle, liver, brain, epidural space, neck, retroperitoneum, and eye.^5–11^ *C. koseri* renal and perinephric abscesses are notably rare, with only four cases reported in the literature.^2,5,12,13^ Here, we report a case of *C. koseri* renal abscess in an elderly patient with prostate cancer and diabetes, who was treated successfully with drain placement and ciprofloxacin.

## Case report

A 70-year-old male with a past medical history of prostate cancer status post-radiation therapy and in remission off treatment since 2010, diabetes, hypertension, and hyperlipidemia, presented to the emergency department for 3 days of right flank pain with intermittent hematuria. He denied dysuria, urinary frequency, urinary urgency, fevers, chills, or night sweats. His medication list included metformin only. Social history was remarkable for one glass of wine per day, and negative for illicit drug use.

On presentation, he had a temperature of 36.9 degrees Celsius, heart rate of 118 beats per minute, blood pressure of 103/61 mm Hg, respiratory rate of 18, oxygen saturation of 100% on room air, and weight of 96.5 kg. His exam was notable for right-sided abdominal tenderness, but negative for suprapubic tenderness or costovertebral angle tenderness. The rest of his exam was unremarkable.

**Figure 1.**
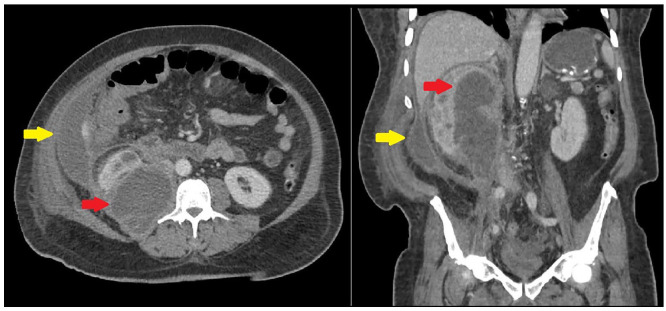
Initial CT showing renal (red arrow) and perihepatic abscesses (yellow arrow).

His initial labs included: white blood cell (WBC) count 16.3 K/cumm (reference range: 4.5–10 K/cumm) with 91% neutrophils, hemoglobin 5.5 g/dL (reference range: 13.5–16.5 g/dL), mean corpuscular volume 70.8 fL (reference range: 82–97 fL), platelets 1,136 K/cumm (reference range: 160–360 K/cumm), sodium 131 mmol/L (reference range: 136–144 mmol/L), creatinine 1.95 mg/dL (reference range: 0.64–1.27 mg/dL) with a baseline of 1.5 mg/dL, glucose 123 mg/dL (reference range: 74–118 mg/dL), albumin 1.8 g/dL (reference range: 3.5–4.8 g/dL), and lactate 2.3 mmol/L (reference range: 0.5–2.2 mmol/L). Urinalysis was remarkable for >50 WBCs/high power field (HPF), 6–10 red blood cells/HPF, and 5–10 granular casts/low power field. Urine culture was negative. His prostate-specific antigen level was 0.2 ng/mL (reference range: 0–4 ng/mL). Renal ultrasound showed a 7.1 × 6.9 × 18 cm complex mixed solid and cystic fluid collection in the right kidney without significant Doppler flow. A contrast-enhanced multiphase computerized tomography (CT) scan of the kidneys (Figure 1) showed a 7.5 × 9.5 × 18 cm rim-enhancing subcapsular fluid collection in the right kidney with extension into the right psoas muscle. In addition, there was a 3.5 × 10 × 10 cm partially rim-enhancing fluid collection in the inferior hepatic region and paracolic gutter.

The patient was admitted to medicine for pyelonephritis, and was given intravenous (IV) fluids, ceftriaxone 1 g every 24 h, and two units of packed red blood cells. Urology and interventional radiology were consulted due to concern for renal hematoma versus renal abscess. The patient’s hemoglobin remained stable; however, his WBC count worsened to 17.2 K/cumm despite antibiotics, so that on hospital day (HD) 3, two separate drains were placed into the renal and perihepatic abscesses under CT guidance. The renal abscess fluid grew *C. koseri* and the perihepatic abscess fluid grew *C. koseri* in addition to *P. mirabilis. C. koseri* was sensitive to ceftriaxone, trimethoprim–sulfamethoxazole, ciprofloxacin, ceftazidime, and gentamicin, but resistant to ampicillin. Ceftriaxone was continued. The fluid cytology was negative for malignant cells.

**Figure 2.**
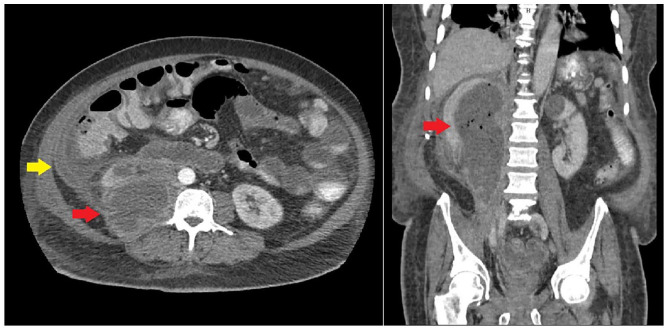
Repeat CT on hospital Day 7 showing improvement of renal abscess (red arrow) and persistence of perihepatic abscess (yellow arrow).

**Figure 3.**
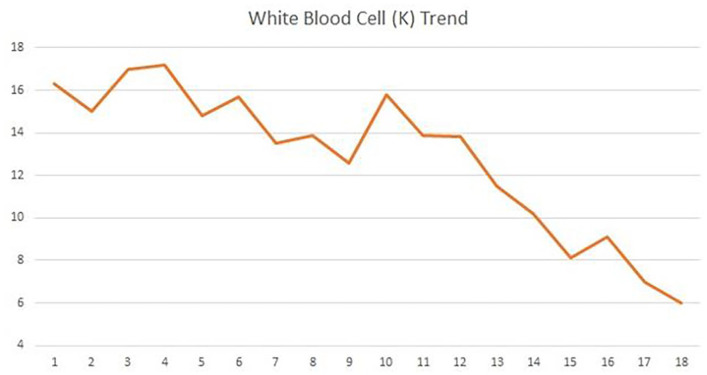
White blood cell (WBC) trend during hospitalization.

A repeat CT scan of the kidneys was performed on HD 7 (Figure 2) due to persistently elevated WBC and diminishing output from both drains; this showed a persistent subcapsular renal abscess measuring 7.5 × 8.5 × 22 cm, and improvement of the perihepatic abscess. On HD 8, interventional radiology exchanged the drain in the renal abscess due to persistence of a large abscess and removed the perihepatic drain. During the following week, the WBC count trended down (Figure 3) but the renal drain continued to have purulent output. On HD 15, a third CT scan showed a persistent, albeit smaller, renal abscess, measuring 5.7 × 2.7 × 14.5 cm, so that, another drain exchange was performed on HD 16. The renal drain continued to have output, but his WBC count normalized, so that, he was discharged on HD 18 (30 January, 2021) with the drain in place, with plan to continue oral ciprofloxacin 500 mg twice a day for 4 weeks, and repeat imaging in 4 weeks. The CT scan done 4 weeks post-hospitalization showed resolution of the abscesses, after which the renal drain was removed. The patient continued to do well a year post-hospitalization without recurrence of the abscesses.

## Discussion

Initial management of a renal abscess consists of antimicrobial therapy and, when indicated, percutaneous drainage. The antibiotic regimen is tailored based on microbiological and susceptibility results. As detailed above, our patient’s renal abscess fluid grew *C. koseri*. In choosing a regimen, it is important to consider inherent or acquired drug resistances. One single-center study demonstrated that approximately 73% of its *Citrobacter* species was multi-drug resistant.^14^ *C. koseri* has been shown to produce both chromosomal AmpC beta-lactamase and extended spectrum beta-lactamase (ESBL).^15^ Antibiotics that have been shown to be effective against *Citrobacter* spp. include third-generation cephalosporins, piperacillin–tazobactam, fluroquinolones, and carbapenems.^16^ Aminoglycosides can also be used.^15^ Our patient was initially treated with ceftriaxone before transitioning to oral ciprofloxacin on discharge from the hospital. The optimal duration of antibiotic therapy can vary depending on clinical response, and abscess drainage may be indicated as discussed below. In a study examining patients with renal abscesses < 5 cm who were treated with antibiotics alone, time to clinical regression and radiographic resolution (based on CT imaging) ranged from 3 to 14 weeks.^17^

The literature indicates that patients with *C. koseri* abscesses in the kidneys, liver, retroperitoneal space, and iliopsoas muscle were treated successfully with abscess drainage in addition to antibiotics.^5,18^ Renal abscesses are generally categorized and managed based on the size of the abscess. Renal abscesses smaller than 3 cm generally respond well to antibiotic treatment alone, while abscesses larger than 5 cm usually require drainage.^19^ Abscesses between 3 and 5 cm are assessed on a case-to-case basis. One study demonstrated 100% success rate using antibiotics alone for management of renal abscesses smaller than 3 cm, while 69% of renal abscesses larger than 5 cm required percutaneous drainage or open surgical intervention.^19^ Factors associated with antibiotic failure include concomitant obstructive uropathy, severe vesicoureteral reflux, diabetes, and old age.^20^ Many patients can be discharged with drains in place if clinical status is improved, as determined by various factors, such as symptom improvement, resolution of fevers, and normalization of WBC count. Outpatient treatment and follow-up often consist of continued oral or IV antibiotics, monitoring of drain output, and repeat imaging.^19^ Our patient had his drain removed 2 months after it was placed when the drain no longer had output and the CT scan showed resolution of the abscess.

Including this one, there are five reported cases of *C. koseri* renal or perinephric abscesses (Table 1).^2,5,12,13^ All five patients were treated successfully with antibiotics alone or in combination with abscess drainage or surgery. In addition, all five patients had at least one co-morbidity that predisposed them to infection with *Citrobacter*, which highlights the opportunistic nature of the bacteria. One patient with *C. koseri* perinephric abscess had an additional complication of empyema, and was treated successfully with needle drainage and ceftazidime.^13^ Another patient with *C. koseri* renal abscess that was complicated by endogenous endophthalmitis was treated successfully with 2 weeks of oral ciprofloxacin and IV ceftriaxone and did not require surgical intervention.^2^ A third patient with *C. koseri* renal abscess was treated with drain placement and 4 weeks of IV ciprofloxacin plus an additional 4 weeks of oral ciprofloxacin.^5^ Finally, the last patient had a perinephric abscess of their transplanted kidney and was treated with an open nephrectomy and washout, in addition to IV gentamicin.^12^

**Table 1.** Reported cases of *Citrobacter* renal abscesses.

Age/gender	Comorbidities	Presentation	Size	Antibiotics	Surgery	Outcome	Reference
70 yo/male	Prostate cancer Diabetes			Ciprofloxacin	Drain placement	Survived	Our patient
52 yo/female	Spina bifida Crossed fused renal ectopia Ileal conduit Nephrolithiasis	5 days of diffuse abdominal pain radiating to right groin	N/A	Ceftazidime	Needle drainage	Survived	13
69 yo/female	Diabetes Recurrent UTIs Stable angina	4 weeks of unresolving UTI that failed nitrofurantoin and cephalexin	2.4 × 2.9 × 2.3 cm	Ciprofloxacin Ceftriaxone	N/A	Survived	2
51 yo/female	Coronary artery disease Diabetes Nephrolithiasis	1 day of flank pain and fevers Discharged with oral antibiotics but was non-complaint Returned with septic shock	4.7 × 7.3 cm	Ciprofloxacin IV × 4 weeks, then PO for 1 week	Drain placement	Survived	5
40 yo/male	Kidney transplant Splenectomy	Admitted for renal transplantation but developed sepsis on post-operative day 11	N/A	Gentamicin	Washout Nephrectomy	Survived	12

This case reiterates that *C. koseri* is an opportunistic organism. Our patient had risk factors for development of *C. koseri* renal abscess, including age, gender, prostate cancer, and diabetes. This case is unique because the patient had two large abscesses with *C. koseri*, he lacked bacteremia despite having systemic manifestations of sepsis with large abscesses, and that he had a complicated course requiring several drains to be treated successfully. Although the patient did not respond optimally to the first drain placement, after drain re-positioning and continued antibiotic therapy, he was ultimately treated successfully with complete resolution of the abscesses. When managing large renal abscesses, it is important to consider a multi-modal approach that includes antibiotics and drainage. Serial WBC and imaging are particularly important with larger abscesses to ensure that repeat drainage is not necessary as was in this case.

## Conclusion

In conclusion, we reported a case of a large renal abscess caused by *C. koseri*, which is an opportunistic organism. It is often treated successfully with antibiotics in addition to drainage depending on the size of the abscess. Generally, renal abscesses that are less than 3 cm in size are treated with antibiotics alone, whereas abscesses greater than 5 cm are treated with antibiotics and drainage. Renal abscesses between 3 and 5 cm are managed with an individualized approach.
